# Planetary Health Diet and Risk of Cardiometabolic Diseases Among Women With Gestational Diabetes

**DOI:** 10.1001/jamanetworkopen.2025.40170

**Published:** 2025-11-07

**Authors:** Xin Yin, Jiaxi Yang, Dong Daniel Wang, Frank B. Hu, Walter C. Willett, Cuilin Zhang

**Affiliations:** 1 Global Centre for Asian Women’s Health, Yong Loo Lin School of Medicine, National University of Singapore, Singapore; 2 Department of Obstetrics and Gynaecology, Yong Loo Lin School of Medicine, National University of Singapore, Singapore; 3 Bia-Echo Asia Centre for Reproductive Longevity and Equality, Yong Loo Lin School of Medicine, National University of Singapore, Singapore; 4 School of Medicine, The Chinese University of Hong Kong, Shenzhen, China; 5 Department of Nutrition, Harvard T. H. Chan School of Public Health, Boston, Massachusetts; 6 Channing Division of Network Medicine, Department of Medicine, Brigham and Women’s Hospital and Harvard Medical School, Boston, Massachusetts; 7 Department of Epidemiology, Harvard T. H. Chan School of Public Health, Boston, Massachusetts

## Abstract

**Importance:**

The Planetary Health Diet (PHD) integrates health and environmental sustainability, yet its association with chronic disease outcomes remains underexplored, particularly among women with a history of gestational diabetes (GD), who are at elevated risk of type 2 diabetes (T2D) and cardiovascular disease (CVD).

**Objective:**

To investigate the associations of adherence to the PHD with risks of T2D, CVD, and long-term weight change among women with a history of GD.

**Design, Setting, and Participants:**

This cohort study used data from the Nurses’ Health Study II. A subset of participants with a history of GD was followed up from June 1991 to June 2021. Data were analyzed from February 1, 2024, to April 9, 2025.

**Exposures:**

Adherence to the PHD was assessed using the Planetary Health Diet Index (PHDI), which was derived from food frequency questionnaires administered every 4 years.

**Main Outcomes and Measures:**

Cox proportional hazards regression models were used to assess hazard ratios (HRs) and 95% CIs for the risk of T2D and CVD. Associations between PHDI changes and concurrent 4-year weight changes were evaluated using multivariable marginal models with generalized estimating equations.

**Results:**

During 120 465 person-years of follow-up among 4633 women with a history of GD (mean [SD] age, 38.9 [6.1] years), 90 incident CVD cases (38 myocardial infarction [MI] and 52 stroke) and 1053 incident T2D cases were observed. Women in the highest tertile of the PHDI had a 63% lower risk of MI (HR, 0.37; 95% CI, 0.16-0.86; *P* for trend = .01) compared with those in the lowest tertile, independent of body mass index (BMI) and other covariates. Higher PHDI was also associated with a lower risk of overall CVD and T2D; however, mediation analysis indicated that BMI accounted for 79.6% (95% CI, 16.5%-98.7%; *P* < .001) of the association with T2D and 15.1% (95% CI, 2.8%-52.5%; *P* = .02) of the association with CVD. Notably, decreasing PHDI was associated with greater weight gain (*P* for trend < .001), with the group experiencing the largest 4-year decrease in PHDI showing a mean weight gain of 2.3 (95% CI, 2.0-2.6) kg.

**Conclusions and Relevance:**

In this cohort study of women with a history of GD, higher PHDI was associated with lower risk of myocardial infarction and better weight management. These findings also emphasize the importance of postpartum weight management to reduce the risk of progression from GD to T2D and CVD.

## Key Points

**Question:**

Is adherence to the new Planetary Health Diet (PHD) pattern associated with lower risks of type 2 diabetes (T2D) and cardiovascular disease (CVD) in women with a history of gestational diabetes (GD)?

**Findings:**

In this cohort study of 4633 women with a history of GD, greater adherence to the PHD was associated with a lower risk of myocardial infarction, while associations with overall CVD and T2D were inverse but largely mediated by body mass index. Additionally, decreasing adherence to the PHD was associated with greater weight gain.

**Meaning:**

These findings suggest that the PHD pattern may play an important role in improving cardiometabolic health and supporting weight management in women with a history of GD.

## Teaser

This cohort study investigates the associations between adherence to the Planetary Health Diet and major cardiometabolic outcomes, including the development of type 2 diabetes, cardiovascular disease (CVD), and long-term weight change among women with a history of gestational diabetes.

## Introduction

In 2019, the EAT*-Lancet* Commission on Healthy Diets From Sustainable Food Systems launched the Planetary Health Diet (PHD) in an effort to enhance the prevention of chronic diseases and promoting environmental sustainability.^1^ Food systems are one of the most important contributors to greenhouse gas emissions,^2^ with agricultural production accounting for 80% to 86% of total food system emissions.^3^ Additionally, two-thirds of fresh water withdrawals were used for irrigation, which returns less water to rivers and groundwater compared with industrial and municipal uses and is the main driver of 90% to 95% of global scarcity–weighted water use.^4^ Consequently, there is an urgent need to transform global diets to benefit both human health and the environment.^1,2^ Recent environmental studies have supported this new diet, highlighting its environmental advantages.^5,6,7^ By aligning nutritional goals with sustainable food production, the PHD offers a unique opportunity to address 2 pressing global challenges simultaneously: improving population health and reducing the environmental footprint of food systems.

Early prevention of type 2 diabetes (T2D) and cardiovascular disease (CVD) is crucial, particularly for populations at high risk, for example, women with a history of gestational diabetes (GD).^8^ GD is widely recognized as a significant risk factor for the subsequent development of T2D and CVD.^9,10^ Research has indicated that women with a history of GD are nearly 10 times more likely to develop T2D later in life than those without a history of GD,^9^ and they also face a 40% increased risk of CVD.^10^ Therefore, identifying modifiable factors is crucial for this high-risk group. Previous studies have demonstrated that adherence to the PHD was associated with a lower risk of T2D, CVD, and all-cause mortality in the general population,^11,12,13^ although findings have been inconsistent, with some studies reporting no associations.^14,15,16^ To our knowledge, no research has yet examined the association between this new dietary pattern and the risk of T2D or CVD among these high-risk women.

Additionally, as obesity is a significant risk factor for both T2D and CVD, weight gain after pregnancy has also been positively associated with a significantly increased risk of progression from GD to T2D and CVD.^17,18^ Whether adherence to the PHD can effectively support weight management in this high-risk population remains unknown. To address these gaps, this study comprehensively examined the associations between adherence to the PHD and major cardiometabolic outcomes, including the development of T2D, CVD, and long-term weight gain in this high-risk population following a pregnancy with a diagnosis of GD.

## Methods

### Study Population

The Nurses’ Health Study II (NHSII) is an ongoing prospective cohort study established in 1989, initially recruiting 116 429 registered female nurses aged 24 to 44 years.^19^ Women with a history of GD from the NHSII were included in the present study. Participants are engaged biennially through self-reported questionnaires to provide updates on health-related behaviors and disease outcomes. The study protocol was approved by the institutional review boards of Brigham and Women’s Hospital and the Harvard T. H. Chan School of Public Health, Boston, Massachusetts, with participants’ consent implied by the return of the questionnaires. We followed the Strengthening the Reporting of Observational Studies in Epidemiology (STROBE) reporting guideline.

The first-year data on diet and lifestyle were collected in 1991, which is the starting point of follow-up in the present study. We included women who reported GD between 1991 and 2001, with the 2001 questionnaire being the last to include GD questions due to most NHSII participants transitioning beyond reproductive age by that time. A prior validation study confirmed that 94% of self-reported GD cases were confirmed by medical records.^20^ Exclusion criteria for the analysis consisted of (1) a history of type 1 diabetes, multiple gestation pregnancies (twin or multiple), or incomplete birth date information; (2) a history of T2D, CVD (myocardial infarction [MI] or stroke), or cancer prior to GD reporting or baseline; or (3) lack of confirmed GD history. The final analytical sample comprised 4633 participants with a history of GD, who were followed up biennially from June 1991 until the return of the 2019 questionnaire (as of June 2021).

### Assessment of the PHD

Participants completed semiquantitative food frequency questionnaires (FFQs) starting in 1991 and subsequently every 4 years. The FFQ assessed the usual intake of various common food items during the past year and has been extensively validated.^21^ The Planetary Health Diet Index (PHDI) was computed in the questionnaire after the first reported GD diagnosis and then for each subsequent FFQ cycle. The PHDI was calculated based on 15 food groups derived from FFQs.^22^ The score ranged from 0 to 140 points, with higher scores reflective of higher adherence to the PHD. Detailed scoring methods for the PHDI components are provided in eMethods and eTable 1 in Supplement 1.

### Ascertainment of T2D and CVD

Participants reporting physician-diagnosed T2D on each biennial questionnaire were mailed a supplemental questionnaire regarding symptoms, diagnostic tests, and hypoglycemic therapy to confirm self-reported diagnoses. Confirmation of T2D required at least 1 of the following criteria, as outlined by the American Diabetes Association^23^: (1) 1 or more classic symptoms consisting of excessive thirst, polyuria, weight loss, hunger, pruritus, or coma plus elevated glucose levels (fasting plasma glucose concentration ≥126 mg/dL or random plasma glucose concentration ≥200 mg/dL [to convert to millimoles per liter, multiply by 0.0555]); (2) no symptoms reported but 2 or more elevated plasma glucose concentrations on more than 1 occasion (plasma glucose concentration ≥126 mg/dL, random plasma glucose concentration ≥200 mg/dL, or 2-hour oral glucose tolerance test ≥200 mg/dL); or (3) treatment with insulin or an oral hypoglycemic agent. Details are included in eMethods in Supplement 1).

Incident CVD was defined as fatal and nonfatal MI and fatal and nonfatal stroke (including ischemic and hemorrhagic stroke). Participants who self-reported a newly diagnosed case of CVD on the questionnaire were asked for permission to access their medical records, which were then reviewed by blinded study investigators to confirm the diagnosis. Nonfatal MI was confirmed using World Health Organization criteria,^24^ and nonfatal stroke was confirmed using National Survey of Stroke criteria.^25^ Deaths were identified through the National Death Index^26^ or reports from next of kin or postal authorities. The cause of death was classified based on autopsy reports, hospital records, or death certificates.

### Assessment of Changes in PHDI and Body Weight

Change in body weight (in kilograms) every 4 years was calculated by subtracting the earlier weight measurement from the more recent one, with negative values indicating weight loss and positive values indicating weight gain. Women with missing data on 4-year body weight changes were excluded from this analysis. Similarly, 4-year changes in PHDI scores were calculated to determine the change in PHDI, where negative values indicate a decrease in adherence to the PHD over 4 years, and positive values indicate an increase. These change values were divided into quintiles, ranging from the smallest values (1) to the largest (5). Additionally, PHDI scores at each cycle were categorized into tertiles (1 = low, 2 = medium, and 3 = high). The 4-year PHDI change in tertile transitions between adjacent cycles were classified into 9 groups: stay low, low to medium, low to high, stay medium, medium to low, medium to high, stay high, high to medium, and high to low. A sensitivity analysis was conducted among participants 65 years or older, as unintentional weight loss may occur in individuals older than 65 years due to factors such as an increase in chronic diseases, gradual loss of muscle and bone mass, reduced physical activity, and metabolic changes in later life.^27,28,29^

### Assessment of Covariates

Detailed assessments of covariates are provided in eMethods in Supplement 1. Race and ethnicity were self-reported in the 1989 baseline questionnaire and were included in the analysis to examine their associations with the PHDI and the risk of CVD and T2D. Because most participants were White, race and ethnicity were categorized as White and other race or ethnicity (including American Indian or Alaska Native, Asian, Black, Native Hawaiian or Other Pacific Islander, and multiracial). Family history of any diabetes and CVD in first-degree relatives was assessed in 1989, 1997, 2001, and 2005. Information on parity, physician-diagnosed illnesses, smoking status, oral contraceptive use, menopausal status, and body mass index (BMI) was collected biennially. Alcohol, sodium, and total energy intake were derived from the FFQ every 4 years. Total physical activity was determined by the frequency of engaging in common recreational activities, from which metabolic equivalent task–hours per week were calculated.^30^ If any covariate was missing from the questionnaire year, the value from the preceding questionnaire was carried forward.

### Statistical Analysis

Data were analyzed from February 1, 2024, to April 9, 2025. Baseline was the questionnaire year when a participant initially reported a pregnancy with GD between 1991 and 2001. The follow-up period was calculated from the date of the GD diagnosis to the earliest of the following events: diagnosis of T2D or CVD, death, last response to the biennial questionnaire, or the end of follow-up June 30, 2021 (corresponding to the latest return of the 2019 questionnaire).

To represent long-term intake and minimize within-person variation, we calculated time-updated cumulative means of dietary data every 4 years, starting from the first post-GD follow-up cycle (ie, baseline) to the start of each follow-up interval (eg, in the 1999 cycle, the cumulative mean PHDI was calculated as the mean of 1991, 1995, and 1999 values). If a woman was pregnant during a subsequent FFQ cycle, dietary data for that year were considered missing, as intake during pregnancy does not accurately reflect her usual long-term dietary pattern. Dietary data were also considered missing if the FFQ was implausible, defined as leaving more than 70 items blank or reporting total energy intake outside the range of 500 to 3500 kcal/d. For cycles with missing or implausible data, values were carried forward from the most recent questionnaire with valid information. Covariates were updated in the same questionnaire cycles as the dietary assessments (ie, every 4 years), with all variables (apart from race and family history) collected before or during the corresponding cycle to retain the prospective nature of the study.

We used Cox proportional hazards regression models to estimate hazard ratios (HRs) and 95% CIs for the association between the cumulative mean PHDI and the risk of T2D or CVD among women with a history of GD. We conducted a likelihood ratio test comparing models with and without the interaction term between exposure and time since diagnosis of GD (<20.6 vs ≥20.6 years [ie, median]). The nonsignificant likelihood ratio tests indicated no violation of the proportional hazards assumption. All Cox proportional hazards models were stratified by age (in months) and calendar time (model 1). In model 2, we further adjusted for parity, race and ethnicity, family history of any diabetes or CVD, oral contraceptive use, menopausal status, cigarette smoking, physical activity, total energy intake, alcohol intake, sodium intake, and ever having hypertension and/or high cholesterol levels. In model 3, we additionally adjusted for BMI. Because BMI is a possible mediator in the pathway between dietary exposure and disease outcomes, it was modeled separately. We conducted mediation analysis using an SAS macro (%mediate; SAS Institute Inc) developed by the Harvard T. H. Chan School of Public Health,^31,32^ which implemented the classic difference-based approach to estimate the proportion of association explained by BMI (treated as a continuous variable), along with 95% CIs and *P* values. We conducted tests of linear trend across categories of the PHDI by assigning the median value for each category and fitting this continuous variable into the models. We also calculated HRs and 95% CIs per IQR increase in PHDI (ie, 15 points). We examined potential nonlinear associations between PHDI and the incidence of CVD and T2D using restricted cubic spline models with 3 knots.^33^ We evaluated potential effect modification by BMI, age, and physical activity on the associations between PHDI and the risks of CVD and T2D by including interaction terms in the regression models and assessing them using the Wald test. For CVD and its subtypes, the PHDI was categorized into tertiles due to the relatively small number of cases, which prevented model convergence when using quartiles or quintiles. In contrast, given the sufficient number of T2D cases, the PHDI was further divided into quintiles for the sensitivity analysis. Additionally, we investigated the association between the intake of each food group in the PHDI (measured both as a score index and in grams) and the risk of T2D or CVD, including CVD subtypes.

We calculated the mean 4-year change in body weight according to concurrent 4-year changes in PHDI. Multivariable marginal models with generalized estimating equations were used to estimate the least squares means of 4-year weight changes and 95% CIs for each category of PHDI change. To account for repeated measures within individuals, an autoregressive variance-covariance matrix was used.

To assess the robustness of our findings, we conducted several sensitivity analyses. First, to minimize potential reverse causation, we repeated the analyses after excluding participants who developed T2D or CVD within the first 4 years of follow-up. Second, among participants with available sleep data, which were collected only from 2001 onward, we further adjusted for mean daily sleep duration.^34^ Third, we derived 6 additional dietary patterns from the FFQ: the Alternate Healthy Eating Index–2010 (AHEI),^35^ Dietary Approaches to Stop Hypertension (DASH),^36^ Alternate Mediterranean Diet (AMED),^37^ Plant-Based Diet Index (PDI), Healthy Plant-Based Diet Index (hPDI), and Unhealthy Plant-Based Diet Index (uPDI).^38^ We compared the PHDI with these dietary patterns using Spearman correlation coefficients. Fourth, we generated cumulative incidence curves to provide an intuitive graphical representation of incidence probabilities. Last, we examined the joint associations between the PHDI and physical activity in relation to CVD and T2D risk. A 2-sided *P* < .05 was considered statistically significant. All statistical analyses were performed using SAS software, version 9.3 (SAS Institute Inc).

## Results

A total of 4633 women with a history of GD (mean [SD] age, 38.9 [6.1] years) were included in this study, contributing 120 465 person-years of follow-up. During a mean (SD) follow-up duration of 25.0 (7.8) years, 90 women developed incident CVD (38 MI and 52 stroke), and 1053 women developed incident T2D. The PHDI scores ranged from a mean (SD) of 58.7 (5.5) in the lowest tertile to 82.5 (6.4) in the highest tertile. A total of 4237 women (91.5%) were White, and 396 (8.5%) were of other race or ethnicity. Participants in the highest PHDI tertile had a lower mean (SD) BMI at baseline, were more likely to have a history of high cholesterol levels and hypertension, engaged in more physical activity, consumed more alcohol, and had lower total calorie and sodium intakes compared with those in the lowest tertile (Table 1).

**Table 1.** Baseline Characteristics of Women With a History of GD According to the PHDI, Nurses’ Health Study II

Characteristic	Tertiles of PHDI (N= 4633)		
	T1 (n = 1544)	T2 (n = 1545)	T3 (n = 1544)
Age, mean (SD), y	37.2 (4.9)	38.5 (5.3)	40.9 (7.1)
Age at GD incidence, mean (SD), y	29.9 (5.0)	30.4 (5.1)	31 (5.4)
Race and ethnicity, No. (%)			
White	1443 (93.5)	1421 (92.0)	1373 (88.9)
Other^a^	101 (6.5)	124 (8.0)	171 (11.1)
BMI at baseline, mean (SD)	27.1 (6.6)	26.6 (6.2)	26.4 (6.2)
Parity, median (IQR)	2 (2-3)	2 (2-3)	2 (2-3)
Family history of diabetes, No. (%)	777 (50.3)	754 (48.8)	755 (48.9)
Family history of CVD, No. (%)	302 (19.6)	278 (18.0)	295 (19.1)
Ever had high cholesterol level, No. (%)	281 (18.2)	273 (17.7)	413 (26.7)
Ever had hypertension, No. (%)	140 (9.1)	140 (9.1)	205 (13.3)
Menopause status, No. (%)			
Premenopausal	1472 (95.3)	1469 (95.1)	1345 (87.1)
Postmenopausal	55 (3.6)	60 (3.9)	156 (10.1)
Other or unknown	17 (1.1)	16 (1.0)	43 (2.8)
Physical activity, mean (SD), MET-h/wk^b^	14.7 (19.9)	17.2 (21.4)	21.0 (29.8)
Smoking status, No. (%)			
Never	1053 (68.2)	984 (63.7)	1034 (67.0)
Past	304 (19.7)	377 (24.4)	388 (25.1)
Current	187 (12.1)	184 (11.9)	122 (7.9)
Oral contraceptives use, No. (%)			
Never	108 (7.0)	121 (7.8)	108 (7.0)
Past	1233 (79.9)	1225 (79.3)	1211 (78.4)
Current	203 (13.1)	199 (12.9)	225 (14.6)
Alcohol intake, mean (SD) g/d	1.9 (4.5)	2.4 (4.8)	2.9 (5.2)
Sodium intake, mean (SD), mg/d	2055 (585)	1877 (565)	1794 (555)
Total energy intake, mean (SD), kcal/d	2399 (779)	2257 (759)	2151 (741)
Baseline PHDI score, mean (SD)	58.7 (5.5)	70.6 (2.6)	82.5 (6.4)
Planetary Health Diet components, mean (SD), g/d			
Whole grains	14 (10)	20 (13)	30 (20)
Starchy vegetables	96 (59)	71 (48)	57 (49)
Vegetables	189 (107)	235 (128)	286 (156)
Whole fruits	101 (84)	140 (101)	190 (130)
Dairy	715 (361)	583 (322)	469 (291)
Red or processed meat	108 (52)	91 (47)	67 (46)
Poultry	58 (36)	53 (34)	47 (39)
Eggs	12 (15)	10 (11)	11 (15)
Fish	18 (18)	27 (23)	35 (30)
Nuts and seeds	4 (5)	5 (5)	9 (14)
Legumes	8 (12)	12 (16)	18 (22)
Soy	0 (3)	1 (5)	8 (44)
Added fat, mean (SD), % of total energy intake			
Unsaturated oils	11 (3)	12 (4)	15 (6)
Saturated oils	9 (3)	8 (3)	7 (3)
Added sugar and fruit juices, mean SD, % of total energy intake	15 (8)	13 (6)	11 (5)

Abbreviations: BMI: body mass index (calculated as weight in kilograms divided by height in meters squared); CVD, cardiovascular disease; GD, gestational diabetes; MET, metabolic equivalent task; PHDI, Planetary Health Diet (PHD) Index.

^a^ Includes American Indian or Alaska Native, Asian, Black, Native Hawaiian or Other Pacific Islander, and multiracial.

^b^ Calculated from the sum of weekly leisure-time moderate- or vigorous-intensity physical activities. 7.5 MET-h/wk is equivalent to 150-min/wk of moderate-intensity physical activity or 75-min/wk of vigorous-intensity physical activity.

Comparing the highest with the lowest tertiles, a higher PHDI was associated with a lower risk of developing CVD after adjusting for confounders, with an HR of 0.55 (95% CI, 0.32-0.97; *P* for trend = .04) (Table 2). This association was mediated by BMI, and further adjustment for BMI resulted in an HR of 0.58 (95% CI, 0.33-1.03; *P* for trend = .06). In the subgroup analysis for MI, after adjusting for covariates, the highest tertile of PHDI was associated with a lower risk of MI, with an HR of 0.35 (95% CI, 0.15-0.83; *P* for trend = .01). After adjusting for BMI, this association resulted in an HR of 0.37 (95% CI, 0.16-0.86; *P* for trend = .01). No association was found between PHDI and the risk of stroke.

**Table 2.** Association Between PHDI and Risks of CVD and T2D Among Women With a History of Gestational Diabetes, Nurses’ Health Study II

Risk factor	Tertiles of PHDI, HR (95% CI)			*P* value for trend	HR (95% CI) per 15-point increment^a^
	T1	T2	T3		
**CVD**					
Cases (n = 90)	37	30	23	NA	NA
Person-years of follow-up	41 686	40 467	38 311	NA	NA
No. of cases/10 000 person-years	8.9	7.4	6.0	NA	NA
Model 1^b^	1 [Reference]	0.76 (0.46-1.23)	0.49 (0.29-0.85)	.01	0.71 (0.53-0.95)
Model 2^c^	1 [Reference]	0.79 (0.48-1.29)	0.55 (0.32-0.97)	.04	0.77 (0.56-1.04)
Model 3^d^	1 [Reference]	0.81 (0.49-1.33)	0.58 (0.33-1.03)	.06	0.79 (0.58-1.09)
**MI**					
Cases (n = 38)	21	10	7	NA	NA
Person-years of follow-up	42 030	40 823	38 646	NA	NA
No. of cases/10 000 person-years	5.0	2.4	1.8	NA	NA
Model 1^b^	1 [Reference]	0.44 (0.22-0.87)	0.27 (0.12-0.60)	<.001	0.49 (0.30-0.78)
Model 2^c^	1 [Reference]	0.49 (0.25-0.99)	0.35 (0.15-0.83)	.01	0.57 (0.35-0.95)
Model 3^d^	1 [Reference]	0.50 (0.25-1.01)	0.37 (0.16-0.86)	.01	0.58 (0.35-0.97)
**Stroke**					
Cases (n = 52)	15	19	18	NA	NA
Person-years of follow-up	41 931	40 666	38 530	NA	NA
No. of cases/10 000 person-years	3.6	4.7	4.4		
Model 1^b^	1 [Reference]	1.14 (0.57-2.32)	0.95 (0.46-1.96)	.86	0.88 (0.60-1.28)
Model 2^c^	1 [Reference]	1.11 (0.55-2.26)	0.97 (0.46-2.04)	.92	0.89 (0.59-1.34)
Model 3^d^	1 [Reference]	1.15 (0.57-2.35)	1.05 (0.50-2.22)	.91	0.93 (0.61-1.41)
**T2D**					
Cases (n = 1053)	415	369	269	NA	NA
Person-years of follow-up	30 849	30 219	28 648	NA	NA
No. of cases/10 000 person-years	134.5	122.1	93.9	NA	NA
Model 1^b^	1 [Reference]	0.84 (0.73-0.97)	0.63 (0.54-0.74)	<.001	0.74 (0.68-0.81)
Model 2^c^	1 [Reference]	0.95 (0.82-1.09)	0.80 (0.68-0.95)	.01	0.85 (0.78-0.93)
Model 3^d^	1 [Reference]	1.01 (0.87-1.17)	0.94 (0.80-1.12)	.53	0.94 (0.86-1.04)

Abbreviations: CVD, cardiovascular disease; HR, hazard ratio; MI, myocardial infarction; NA, not applicable; PHDI, Planetary Health Diet Index; T2D, type 2 diabetes.

^a^ HRs (95% CIs) were calculated per 1-IQR increase in PHDI (ie, 15 points).

^b^ Stratified by age (in months) and calendar time.

^c^ Additionally adjusted for parity (1 or ≥2), race and ethnicity (White or other), family history of any diabetes and/or CVD (yes or no), oral contraceptive use (never, past, or current), menopausal status (premenopausal, postmenopausal, or unsure), cigarette smoking (current, former, or never), physical activity (continuous, in metabolic equivalent task–h/wk), total energy intake (continuous, in kcal/d), alcohol intake (continuous, in g/d), sodium intake (continuous, in mg/d), ever had hypertension (yes or no), and ever had high cholesterol level (yes or no).

^d^ Additionally adjusted for body mass index (continuous).

Comparing the highest with the lowest tertiles, a higher PHDI was initially associated with a reduced risk of developing T2D, with an HR of 0.80 (95% CI, 0.68-0.95; *P* for trend = .01) (Table 2). However, after further adjusting for BMI, the HR was 0.94 (95% CI, 0.80-1.12; *P* for trend = .53). Consistent findings were observed when the PHDI was categorized into quintiles (eTable 2 in Supplement 1). In the mediation analysis of the association between the PHDI and the risk of T2D or CVD, BMI was estimated to mediate 79.6% (95% CI, 16.5%-98.7%; *P* < .001) of the association with T2D risk and 15.1% (95% CI, 2.8%-52.5%; *P* = .02) of the association with CVD risk.

**Figure 1.**
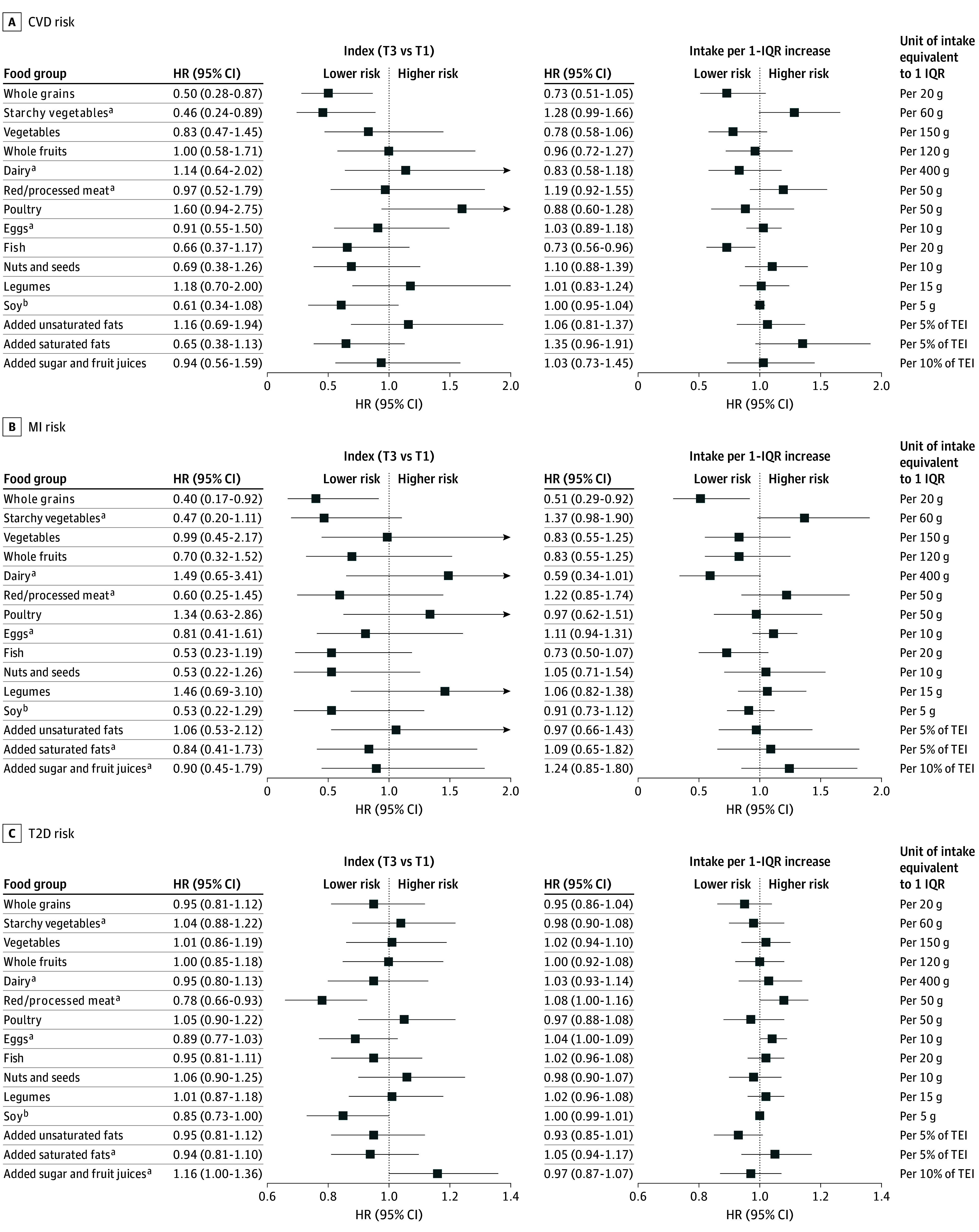
Hazard Ratios (HRs) for Each Food Component of the Planetary Health Diet Index for Cardiovascular Disease (CVD), Myocardial Infarction (MI), and Type 2 Diabetes (T2D) Risk Among Women With a History of Gestational Diabetes TEI indicates total energy intake. ^a^A higher score represents lower consumption because the scores for these unhealthy food groups were reverse coded. ^b^Given the limited variation in soy food consumption, the data were divided into 2 groups, and HRs were calculated for intake versus nonintake.

When assessing the food components of the PHDI, higher intakes of whole grains and fish were associated with a lower risk of CVD, whereas a higher intake of starchy vegetables was associated with an increased risk of CVD (Figure 1 and eTable 3 in Supplement 1). Similarly, a 1-IQR increase in whole grain intake (equivalent to 20 g) was associated with a 49% lower risk of MI (HR, 0.51; 95% CI, 0.29-0.92). Additionally, higher intake of red and processed meat (index tertile 3) was associated with an increased risk of T2D (HR, 0.78; 95% CI, 0.66-0.93), whereas individuals who consumed soy foods had a lower risk compared with those who did not consume any (HR, 0.85; 95% CI, 0.73-1.00).

**Figure 2.**
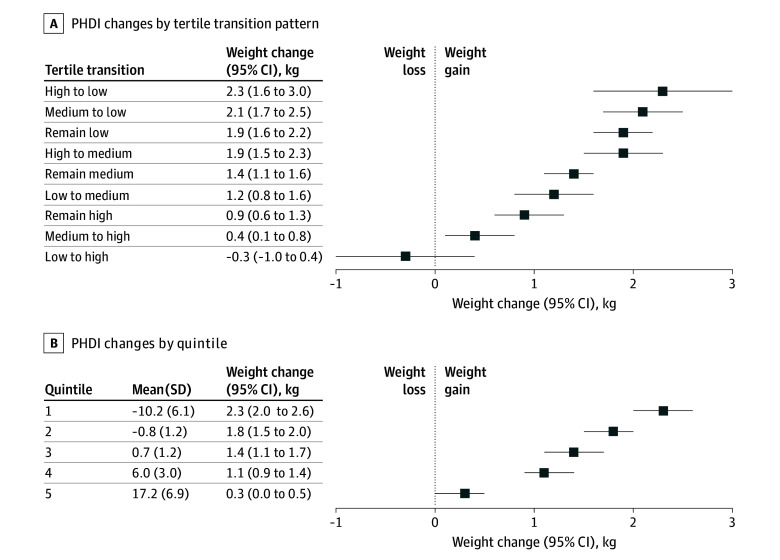
Association Between 4-Year Change in Planetary Health Diet Index (PHDI) and Least Squares Means of Weight Change Among Women With a History of Gestational Diabetes

In this study, 4308 participants provided data on 4-year body weight changes. During the follow-up period, these participants experienced increases in both their PHDI scores and body weight. The mean (SD) change in PHDI across all periods was an increase of 2.7 (10.3), and the mean (SD) weight change was a gain of 1.4 (7.4) kg. An increasing PHDI was associated with lower weight gain, with a *P* for trend < .001 (Figure 2). The group with the highest increase in PHDI (quintile 5, mean [SD], 17.2 [6.9]) had the lowest weight gain, with a mean of 0.3 (95% CI, 0.02-0.5) kg, while the group with the greatest decrease in PHDI (quintile 1, mean [SD], −10.2 [6.1]) experienced the highest weight gain, with a mean of 2.3 (95% CI, 2.0-2.6) kg. When PHDI change was in tertile transitions, the group experiencing the largest decrease in PHDI (from the highest to lowest tertile) showed the greatest weight gain, with a mean weight gain of 2.3 (95% CI, 1.6-3.0) kg. Similar results were observed when the analysis was restricted to participants 65 years or older (eFigure 1 in Supplement 1).

Comparisons of the PHDI with other dietary patterns (AHEI, DASH, AMED, PDI, hPDI, and uPDI) revealed moderate correlations (eTable 4 in Supplement 1). Sensitivity analyses excluding participants who developed CVD or T2D within the first 4 years of follow-up (eTable 5 in Supplement 1) or further adjusting for mean daily sleep duration (eTable 6 in Supplement 1) yielded consistent results. Cumulative incidence curves illustrated the incidence probabilities across PHDI categories (eFigure 2 in Supplement 1). We also examined the joint associations between PHDI and physical activity (eTable 7 in Supplement 1). Compared with women who had both low physical activity and low PHDI scores, those with both high physical activity and high PHDI scores had substantially lower risks of CVD, particularly MI, and T2D (eTable 7 in Supplement 1). No statistically significant interactions were observed between PHDI and BMI, age, or physical activity. Restricted cubic spline analyses indicated no evidence of nonlinearity between PHDI and the risks of CVD or T2D.

## Discussion

In this cohort study of women with a history of GD, during a mean follow-up duration of 25.0 years, higher adherence to the PHD was associated with a lower risk of overall CVD, particularly MI, and T2D. However, the associations with overall CVD and T2D were largely mediated by BMI. Furthermore, greater adherence to the PHD was associated with better weight management, as higher PHDI was associated with less weight gain, which may have subsequently contributed to reduced risks of T2D and CVD.

Because the PHD was only proposed within the last 5 years, the number of studies examining its association with T2D is limited, and the findings are inconsistent.^12,15,39,40,41^ Of the 5 studies conducted across different countries, 4 reported that adherence to the PHD was associated with a reduced risk of T2D,^12,39,40,41^ while 1 study including women in Mexico found no association with T2D.^15^ However, all of these studies were conducted in the general population, and none assessed this association among women at high risk, such as those with a history of GD. There is an urgent need to identify modifiable factors to prevent or delay the onset of T2D and its comorbidities in this population. Our study focused on this high-risk population, and our findings are initially consistent with most previous studies, showing that a higher PHDI was associated with a reduced risk of developing T2D. However, this association was attenuated after adjusting for BMI, and our mediation analysis further confirmed that BMI acted as a significant mediator. Similarly, previous research in the general population^12^ indicated that 44% of the association between PHDI and T2D was mediated by BMI. In our study, we found an even greater mediation effect, with BMI estimated to mediate 79.6% of the association in women with a history of GD. These findings underscore the importance of weight management in reducing the risk of progression from GD to T2D. However, the wide 95% CI (16.5%-98.7%) indicated considerable statistical uncertainty, and the magnitude of this mediation effect should therefore be interpreted with caution. Future research, when methodologically feasible, could extend our analysis by incorporating longitudinal weight change (eg, 4-year weight change) as a mediator, using advanced mediation approaches that account for time-varying exposure, mediator, and outcome data.

Improvements in diet quality were related to better weight management among parous women, particularly those with a history of GD.^42^ Our study found a positive association between long-term adherence to the PHD and favorable weight management, with higher PHDI associated with lower weight gain. Similarly, in the Danish Diet, Cancer, and Health cohort, adherence to the PHD was associated with a lower risk of obesity and reduced waist circumference during the follow-up period,^40^ findings consistent with adherence to the PHD and the lower BMI observed in the European Prospective Investigation into Cancer and Nutrition (EPIC)–Oxford cohort study.^41^ Supported by these results, our study underscores the clinical importance of maintaining a healthy diet and highlights its crucial role in postpartum weight control for this high-risk population.

In the general population, previous studies^11,14,43,44^ have reported conflicting associations between the PHD and the risk of CVD.^11,14,43,44^ Some studies have reported that adherence to the PHD was associated with better cardiovascular health among European adolescents,^11^ as well as lower blood pressure and favorable metabolic biomarkers among adults in Brazil.^43^ In contrast, no association between the PHD and CVD was observed in the UK Biobank study^44^ or the prospective NutriNet-Santé cohort.^14^ No prior research, to our knowledge, has specifically investigated the association between this dietary pattern and CVD risk among women with a history of GD. Our study addressed this gap, finding an association between higher PHDI and a lower risk of overall CVD and MI in this high-risk population, but no association with stroke. Our findings closely align with the results of the EPIC-Oxford study,^41^ which assessed both CVD and T2D in more than 46 000 participants and reported that adherence to the PHD was associated with lower risks of ischemic heart disease and T2D, but not stroke.^41^ In fact, most previous studies in the general population have similarly reported no association between PHDI and stroke.^16,41,45^ This null finding may reflect limited statistical power due to the small number of stroke cases in our cohort, as well as potential biological differences in the pathogenesis of stroke compared with other cardiovascular outcomes (eg, MI). Future studies with larger numbers of stroke cases and more detailed stroke subtyping are warranted to clarify these associations.

Compared with other established dietary patterns (eg, AHEI, AMED, DASH, PDI, hPDI, and uPDI), the unique strength and novelty of the PHD lie in its explicit emphasis on environmental sustainability through stricter limits on certain animal-based foods, especially red and processed meats, given their substantially higher greenhouse gas emissions compared with plant-based alternatives.^4,46^ Furthermore, unlike the PDI, which negatively scores all animal-based foods, the PHDI positively scores selected animal-based foods, such as fish, recognizing their lower environmental footprint and potential long-term health benefits. At the same time, the PHDI uniquely penalizes specific plant-based foods (eg, starchy vegetables such as potatoes), reflecting their relatively higher environmental impact due to greater irrigation demands and increased inputs of fertilizers and pesticides, which contribute to soil degradation and water pollution.^4,46,47^ Given the recent development of the PHDI, few prior studies have specifically evaluated this dietary pattern within high-risk populations, such as women with prior GD. Therefore, our findings provide critical evidence supporting the adoption of the PHDI as an actionable dietary strategy that simultaneously promotes personal health and planetary health, aligning with contemporary public health priorities emphasizing integrated health-environment interventions.

In our study, the primary dietary components driving the protective associations between the PHDI and reduced risks of CVD and T2D included higher intake of whole grains, fish, and soy, coupled with limited consumption of starchy vegetables and red and processed meats. Consistent with previous findings, whole grain consumption was associated with a lower risk of CVD, reduced inflammation, and improved weight management.^48,49^ Similarly, regular fish consumption, even at modest levels, provided ω-3 fatty acids and substantially reduced cardiovascular mortality.^50,51^ Conversely, high intake of red and processed meats was associated with an increased risk of T2D,^52^ whereas replacing red meat with plant-based proteins (eg, legumes, nuts, and soy) improved blood lipid profiles.^53^ Soy consumption has also been associated with improved cardiometabolic health among women.^54^ Last, consistent with existing evidence, we observed that higher consumption of starchy vegetables was associated with an increased risk of CVD. This may be explained by their high glycemic load, which has been linked to greater weight gain^55^ and increased cardiovascular risk.^56,57^

### Strengths and Limitations

Our study has several unique strengths. To the best of our knowledge, this is the first study to assess the association between the PHD and the risks of T2D, CVD, and weight gain among women with a history of GD. Additionally, the prospective cohort design, with its long follow-up period and large sample size of women with a history of GD, substantially strengthens the validity of our findings. However, this study has some limitations. First, participants self-reported dietary information, which may introduce measurement error. However, the repeated assessments of diet quality and lifestyle factors every 4 years, along with the use of cumulative mean measurements, help mitigate random errors in exposure measurements. Second, despite adjusting for numerous potential confounders, the possibility of residual or unmeasured confounding cannot be entirely ruled out. However, as all participants were registered nurses with similar educational attainment and occupation, this likely reduced bias. Third, the generalizability of our findings may be limited, as most participants were White; other racial and ethnic groups may have different dietary patterns or may not have consumed certain foods in the PHDI.^58^ Further prospective cohort studies are needed to validate our findings in other racial and ethnic groups.

## Conclusions

In this cohort study of women with a history of GD, we found that greater adherence to the environmentally sustainable PHD was associated with a lower risk of overall CVD, particularly MI, and T2D. However, the associations with overall CVD and T2D were largely mediated by BMI, highlighting the key role of weight management in reducing long-term cardiometabolic risks. Higher PHDI was associated with better weight management (ie, less weight gain). These findings emphasize the importance of promoting healthy dietary patterns and postpartum weight management to reduce the long-term risk of T2D and CVD in this high-risk population. Incorporating structured dietary counseling based on the PHD into routine postpartum follow-up for women with prior GD may offer a practical approach to lowering future cardiometabolic risk while advancing environmental sustainability objectives.

## Notes

Supplement 1. **eMethods.** Population, Planetary Health Diet, Ascertainment of T2D and CVD, Assessments, and Analysis **eTable 1.** Planetary Health Diet Index **eTable 2.** Association Between PHDI and the Risk of T2D Among Women With a History of GD, Nurses’ Health Study II **eTable 3.** Hazard Ratios (95% CIs) for Each Food Component of the PHDI for CVD and T2D Risk Among Women With a History of GD, Nurses’ Health Study II **eTable 4.** Spearman Correlation Coefficients Between the Cumulative Mean PHDI and Other Dietary Patterns, Nurses’ Health Study II **eTable 5.** Association Between PHDI and Risks of CVD and T2D Among Women With a History of GD, Excluding Cases in the First 4 Years of Follow-Up, Nurses’ Health Study II **eTable 6.** Association Between PHDI and Risks of CVD and T2D Among Women With a History of GD and Available Sleep Duration (n = 3660), Nurses’ Health Study II **eTable 7**. Joint Association of PHDI and Physical Activity With Risks of CVD and T2D Among Women With a History of GD, Nurses’ Health Study II **eTable 8.** Association Between 4-Year Change in PHDI and Least Squares Means of Weight Change (kg) Among Women With a History of GD, Nurses’ Health Study II **eFigure 1.** Association Between 4-Year Change in PHDI and Least Squares Means of Weight Change (kg) Among Participants ≤65 Years, Nurses’ Health Study II **eFigure 2.** Cumulative Incidence of T2D and CVD by Tertiles of PHDI Among Women With a History of GD, Nurses’ Health Study II
eReferences

Supplement 2.
Data Sharing Statement
