# Supplementary material for: Planetary Health Diet and Risk of Cardiometabolic Diseases Among Women With Gestational Diabetes
Source: JAMA Netw Open. 2025 Nov 7;8(11):e2540170. doi: 10.1001/jamanetworkopen.2025.40170 (PMC12595533; doi:10.1001/jamanetworkopen.2025.40170)
Supplement: Supplement 1. — eMethods. Population, Planetary Health Diet, Ascertainment of T2D and CVD, Assessments, and Analysis eTable 1. Planetary Health Diet Index eTable 2. Association Between PHDI and the Risk of T2D Among Women With a History of GD, Nurses’ Health Study II eTable 3. Hazard Ratios (95% CIs) for Each Food Component of the PHDI for CVD and T2D Risk Among Women With a History of GD, Nurses’ Health Study II eTable 4. Spearman Correlation Coefficients Between the Cumulative Mean PHDI and Other Dietary Patterns, Nurses’ Health Study II eTable 5. Association Between PHDI and Risks of CVD and T2D Among Women With a History of GD, Excluding Cases in the First 4 Years of Follow-Up, Nurses’ Health Study II eTable 6. Association Between PHDI and Risks of CVD and T2D Among Women With a History of GD and Available Sleep Duration (n = 3660), Nurses’ Health Study II eTable 7. Joint Association of PHDI and Physical Activity With Risks of CVD and T2D Among Women With a History of GD, Nurses’ Health Study II eTable 8. Association Between 4-Year Change in PHDI and Least Squares Means of Weight Change (kg) Among Women With a History of GD, Nurses’ Health Study II eFigure 1. Association Between 4-Year Change in PHDI and Least Squares Means of Weight Change (kg) Among Participants ≤65 Years, Nurses’ Health Study II eFigure 2. Cumulative Incidence of T2D and CVD by Tertiles of PHDI Among Women With a History of GD, Nurses’ Health Study II eReferences [file jamanetwopen-e2540170-s001.pdf]

Supplementary Online Content

Yin X, Yang J, Wang DD, Hu FB, Willet WC, Zhang C. Planetary health diet and risk of cardiometabolic diseases among women with gestational diabetes. *JAMA Netw Open.* 2025;8(10):e2540170. doi:10.1001/jamanetworkopen.2025.40170

eMethods. Population, Planetary Health Diet, Ascertainment of T2D and CVD, Assessments, and Analysis

eTable 1. Planetary Health Diet Index

eTable 2. Association Between PHDI and the Risk of T2D Among Women With a History of GD, Nurses' Health Study II

eTable 3. Hazard Ratios (95% CIs) for Each Food Component of the PHDI for CVD and T2D Risk Among Women With a History of GD, Nurses' Health Study II

eTable 4. Spearman Correlation Coefficients Between the Cumulative Mean PHDI and Other Dietary Patterns, Nurses' Health Study II

eTable 5. Association Between PHDI and Risks of CVD and T2D Among Women With a History of GD, Excluding Cases in the First 4 Years of Follow-Up, Nurses' Health Study II

eTable 6. Association Between PHDI and Risks of CVD and T2D Among Women With a History of GD and Available Sleep Duration (n = 3660), Nurses' Health Study II

eTable 7. Joint Association of PHDI and Physical Activity With Risks of CVD and T2D Among Women With a History of GD, Nurses' Health Study II

eTable 8. Association Between 4-Year Change in PHDI and Least Squares Means of Weight Change (kg) Among Women With a History of GD, Nurses' Health Study II

eFigure 1. Association Between 4-Year Change in PHDI and Least Squares Means of Weight Change (kg) Among Participants ⩽65 Years, Nurses' Health Study II

eFigure 2. Cumulative Incidence of T2D and CVD by Tertiles of PHDI Among Women With a History of GD, Nurses' Health Study II

eReferences

This supplementary material has been provided by the authors to give readers additional information about their work.

© 2025 Yin X et al. *JAMA Network Open*.

eMethods. Population, Planetary Health Diet, Ascertainment of T2D and CVD, Assessments, and Analysis

Study Population

The Nurses' Health Study II (NHSII) is an ongoing prospective cohort study established in 1989, initially recruiting 116,429 registered female nurses aged 24-44 years [1]. Women with a history of gestational diabetes mellitus (GD) from NHSII were included in this current study. Participants are engaged biennially through self-reported questionnaires to provide updates on health-related behaviors and disease outcomes. The study protocol was approved by the institutional review boards of the Brigham and Women's Hospital and the Harvard T.H. Chan School of Public Health, with participants' consent implied by the return of the questionnaires. We followed the Strengthening the Reporting of Observational Studies in Epidemiology (STROBE) reporting guideline.

The first-year data on diet and lifestyle were collected were in 1991, which is the start timepoint of follow-up in this current study. We included women who reported GD between 1991 and 2001, with the 2001 questionnaire being the last to include GD questions due to most NHSII participants transitioning beyond reproductive age by that time. A prior validation study confirmed that 94% of self-reported GD cases were confirmed by medical records [2]. Exclusion criteria for the analysis included: (1) a history of type 1 diabetes, multiple gestation pregnancies (twins or multiples), or incomplete birth date information; (2) a history of type 2 diabetes, cardiovascular disease (myocardial infarction or stroke), or cancer prior to GD reporting or baseline; or (3) lack of confirmed GD history. The final analytical sample comprised 4,633 participants with a history of GD, who were followed biennially from 1991 until the return of the 2019 questionnaire (as of June 2021).

Assessment of Planetary Health Diet

Participants completed semiquantitative Food Frequency Questionnaires (FFQs) starting in 1991 and subsequently every 4 years (i.e., 1991, 1995, 1999, 2003, 2007, 2011, 2015, and 2019). The FFQ assessed the usual intake of various common food items over the past year and has been extensively validated [3]. The Planetary Health Diet Index (PHDI) was computed in the questionnaire after the first reported GD diagnosis, and then for each subsequent FFQ cycle. If a woman was pregnant during a subsequent FFQ cycle, the dietary

© 2025 Yin X et al. *JAMA Network Open*.

data for that year was considered missing. This is because dietary intake during pregnancy does not accurately reflect the long-term dietary patterns typically followed by the individual. If participants provided implausible FFQ information, defined as leaving more than 70 items blank or reporting total energy intake outside the range of 500 to 3500 kcal/day, the dietary data for that year was also considered missing.

The PHDI was calculated based on 15 food groups derived from FFQs. Detailed scoring methods for the PHDI components are provided in **eTable 1 in Supplement 1**. Whole grains, vegetables (not including starchy vegetables), whole fruits, poultry (e.g., duck, goose, ostrich), fish, nuts and seeds (e.g., peanuts, tree nuts, such as walnuts, almonds, hazelnuts, pecan, cashews, pistachios), non-soy legumes (e.g., dry beans, lentils, peas), soybeans/soy foods, and added unsaturated fat (not including *trans* fat; e.g., olive soybean, rapeseed, sunflower, peanut oil) were positively scored. Starchy vegetables (e.g., potatoes, cassava), dairy (e.g., milk, cheese, yogurt), red/processed meat (e.g., beef, lamb, pork), eggs, added saturated fat (e.g., palm oil, coconut oil, dairy fat (butter), margarine, lard, tallow), and added sugar and fruit juices were negatively scored. The score ranges from 0 to 140 points, where a higher score is reflective of higher adherence to the PHD.

Ascertainment of T2D and CVD

Participants reporting physician diagnosed T2D on each biennial questionnaire were mailed a supplemental questionnaire regarding symptoms, diagnostic tests, and hypoglycemic therapy to confirm self-reported diagnoses. Confirmation of diabetes required at least one of the following criteria, as outlined by the American Diabetes Association (ADA) [4]: (1) one or more classic symptoms (excessive thirst, polyuria, weight loss, hunger, pruritus, or coma) plus elevated glucose levels (fasting plasma glucose concentration ≥ 7.0 mmol/L or random plasma glucose ≥ 11.1 mmol/L); or (2) no symptoms reported but two or more elevated plasma glucose concentrations on more than one occasion (fasting ≥ 7.0 mmol/L, random ≥ 11.1 mmol/L, 2-hour oral glucose tolerance test ≥ 11.1 mmol/L); or (3) treatment with insulin or oral hypoglycemic agent. Prior to 1998, the diagnosis of diabetes used the National Diabetes Data Group criteria, which required a fasting plasma glucose concentration ≥ 7.8 mmol/L instead of ≥ 7.0 mmol/L [5].

© 2025 Yin X et al. *JAMA Network Open*.

Incident CVD was defined as fatal and non-fatal myocardial infarction (MI), and fatal and non-fatal stroke (including ischemic and hemorrhagic stroke). Participants who self-reported a newly diagnosed case of CVD on the questionnaire were asked for permission to access their medical records, which were then reviewed by blinded study investigators to confirm the diagnosis. Non-fatal MI was confirmed using World Health Organization criteria [6], and non-fatal stroke was confirmed using National Survey of Stroke criteria [7]. Deaths were identified through the National Death Index [8], or reports from next of kin or postal authorities. The cause of death was classified based on autopsy reports, hospital records, or death certificates.

Assessment of Changes in PHDI and Body Weight

Change in body weight (in kilograms, kg) every 4 years was calculated by subtracting the earlier weight measurement from the more recent one, with negative values indicating weight loss and positive values indicating weight gain. Women with missing data on four-year body weight changes were excluded from this analysis, leaving a total of 4,308 participants. Similarly, 4-year changes in PHDI scores were calculated to determine the change in PHDI, where negative values indicate a decrease in adherence to the PHDI over 4 years, and positive values indicate an increase. These change values were divided into quintiles, ranging from the smallest values (Q1) to the largest (Q5). Additionally, PHDI scores at each cycle were categorized into tertiles (T1 = low, T2 = medium, and T3 = high). The 4-year PHDI change in tertile transitions between adjacent cycles were classified into nine groups: stay low, low to medium, low to high, stay medium, medium to low, medium to high, stay high, high to medium, and high to low. A sensitivity analysis was conducted among participants aged ≤65 years, as unintentional weight loss may occur in individuals older than 65 due to factors such as an increase in chronic diseases, gradual loss of muscle and bone mass, reduced physical activity, and metabolic changes in later life [9-11].

Assessment of Covariates

Race and ethnicity were self-reported in the 1989 baseline questionnaire and were included in the analysis to examine their associations with PHDI and the risk of CVD and T2D. Because most participants were White, race and ethnicity were categorized as White and Other (which included American Indian/Native American,

Asian, Black, Hawaiian, and multiracial). Family history of diabetes and CVD in first-degree relatives were assessed in 1989, 1997, 2001, and 2005. Information on parity, physician-diagnosed illnesses, smoking status, oral contraceptive use, menopausal status, and body mass index (BMI) was collected biennially. Parity was defined as the number of pregnancies lasting greater than 6 months. BMI was calculated as weight in kilograms divided by the square of height in meters (kg/m^2). Participants were also asked about physician-diagnosed illnesses, including whether they had ever had high blood pressure (not pregnancy-related) and whether they had ever elevated cholesterol. Alcohol intake (g/d), sodium intake (continuous, mg/d) and total energy intake (kcal/d) were derived from the FFQ every 4 years. Total physical activity was assessed every 4 years, except for one longer interval between 1991 and 1997, and was determined by the frequency of engaging in common recreational activities, from which metabolic equivalent task (MET)-hours per week were calculated [12]. If any covariate was missing from the questionnaire year, the value from the preceding questionnaire was carried forward.

Statistical Analysis

Baseline was the questionnaire year when a participant initially reported a pregnancy with GD between 1991 and 2001. The follow-up period was calculated from the date of the GD diagnosis to the earliest of the following events: diagnosis of T2D or CVD, death, last response to the biennial questionnaire, or the end of follow-up in June 2021 (corresponding to the latest return of the 2019 questionnaire).

To represent long-term intake and minimize within-person variation, we calculated time-updated cumulative averages of dietary data every 4 years, starting from the first post-GD follow-up cycle (i.e., baseline) up to the start of each follow-up interval (e.g., in the 1999 cycle, the cumulative average of PHDI was calculated as the average of 1991, 1995, and 1999 values). If a woman was pregnant during a subsequent FFQ cycle, dietary data for that year were considered missing, as intake during pregnancy does not accurately reflect her usual long-term dietary pattern. Dietary data were also considered missing if the FFQ was implausible, defined as leaving more than 70 items blank or reporting total energy intake outside the range of 500–3,500 kcal/day. For cycles with missing or implausible data, values were carried forward from the most recent questionnaire

© 2025 Yin X et al. *JAMA Network Open*.

with valid information. Covariates were updated in the same questionnaire cycles as the dietary assessments (i.e., every 4 years), with all variables (apart from race and family history) collected before or during the corresponding cycle to retain the prospective nature of the study.

We used Cox proportional hazard models to estimate hazard ratios (HRs) and 95% confidence intervals (CIs) for the association between the cumulative average of PHDI and T2D or CVD risk among women with a history of GD. We conducted a likelihood ratio test comparing models with and without the interaction term between exposure and time since diagnosis of GD (< v ≥20.6 years (i.e., median)). The non-significant likelihood ratio tests indicated no violation of the proportional hazards assumption. All Cox models were stratified by age (in months) and calendar time (Model 1). In Model 2, we further adjusted for parity (1 vs ≥ 2), race/ethnicity (White vs non-White), family history of diabetes or CVD (yes or no), oral contraceptive use (never, past, current), menopausal status (premenopausal, postmenopausal, unsure), cigarette smoking (current, former, never), physical activity (continuous, MET-hours/week), total energy intake (continuous, kcal/day), alcohol intake (continuous, g/day), sodium intake (continuous, mg/day), ever had hypertension (yes or no), and ever had high cholesterol (yes or no). In Model 3, we additionally adjusted for BMI (continuous, kg/m^2). Because BMI is a possible mediator in the pathway between dietary exposure and disease outcomes, it was modeled separately. We conducted mediation analysis using a SAS macro (%mediate) developed by the Harvard T.H. Chan School of Public Health [13,14], which implemented the classical difference-based approach to estimate the proportion of association explained by BMI (treated as a continuous variable), along with 95% CIs and p-values. This macro has been widely applied in the NHS and NHSII cohorts [14,15]. We conducted tests of linear trend across categories of the PHDI by assigning the median value for each category and fitting this continuous variable into the models. We examined potential nonlinear associations between PHDI and the incidence of CVD and T2D using restricted cubic spline models with three knots [16]. We evaluated potential effect modification by BMI, age, and physical activity on the associations between PHDI and the risks of CVD and T2D by including interaction terms in the regression models and assessing them using the Wald test. For CVD and its subtypes, the PHDI was categorized into tertiles due to the relatively small number of cases, which prevented model convergence when using quartiles or quintiles. In contrast,

© 2025 Yin X et al. *JAMA Network Open*.

given the sufficient number of T2D cases, the PHDI was further divided into quintiles for the sensitivity analysis. Additionally, we investigated the association between the intake of each food group in the PHDI (measured both as a score index and in grams) and the risk of T2D or CVD, including CVD subtypes.

We calculated the mean 4-year change in body weight according to concurrent 4-year changes in PHDI. Multivariable marginal models with generalized estimating equations (GEE) were used to estimate the least squares means of 4-year weight changes and 95% confidence intervals (CI) for each category of PHDI change. To account for repeated measures within individuals, an autoregressive variance-covariance matrix was employed.

To assess the robustness of our findings, we conducted several sensitivity analyses. First, to minimize potential reverse causation, we repeated the analyses after excluding participants who developed T2D or CVD within the first four years of follow-up. Second, among participants with available sleep data, which were collected only from 2001 onwards, we further adjusted for average daily sleep duration [17]. Third, we derived six additional dietary patterns from the FFQ: the Alternate Healthy Eating Index-2010 (AHEI) [18], Dietary Approaches to Stop Hypertension (DASH) [19], Alternate Mediterranean Diet (AMED) [20], Plant-based Diet Index (PDI), Healthy Plant-based Diet Index (hPDI), and Unhealthy Plant-based Diet Index (uPDI) [21]. We compared the PHDI with these dietary patterns using Spearman correlation coefficients. Fourth, we generated cumulative incidence curves to provide an intuitive graphical representation of incidence probabilities. Finally, we examined the joint associations between PHDI and physical activity in relation to CVD and T2D risk. A two-sided $P < 0.05$ was considered statistically significant. All statistical analyses were performed using SAS software (version 9.3; SAS Institute Inc.).

© 2025 Yin X et al. *JAMA Network Open*.

eTable 1. Planetary Health Diet Index

	**EAT- LANCET REFERENCE DIET (FOR 2500 KCAL/D)**		**PHDI SCORING CRITERIA (TOTAL 0-140)**		
	grams/day	kcal/day	Min score (0) in grams/day	Max score (10) in grams/day	Weight in score
Whole grains	232 (0-60% of TEI)	811	0	≥ 75 for women<br>≥ 90 for men	1
Starchy vegetables (e.g., potatos, cassava)	50 (0-100)	39	≥200	≤50	1
Vegetables (not including potato or other starchy vegetables)	300 (200-600)	78	0	≥300	1
Whole fruit	200 (100-300)	126	0	≥200	1
Dairy foods (e.g., milk, cheese, yogurt)	250 (0-500)	153	≥1000	≤250	1
Red/processed meat (e.g., beef, lamb, pork)	14 (0-28)	30	≥100	≤14	1
Chicken and poultry (e.g., duck, goose, ostrich)	29 (0-58)	62	≥100	≤29	1
Eggs	13 (0-25)	19	≥120	≤13	1
Fish and shellfish	28 (0-100)	40	0	≥28	1
Nuts (e.g., peanuts, tree nuts, such as walnuts, almonds, hazelnuts, pecan, cashews, pistachios)	50 (0-75)	291	0	≥50	1
Non-soy legumes (e.g., dry beans, lentils, peas)	50 (0-100)	172	0	≥100	0·5
Soybeans/soy foods	25 (0-50)	112	0	≥50	0·5
Added fat – unsaturated oils (not including *trans* fat; e.g., olive soybean, rapeseed, sunflower, peanut oil)	40 (20-80)	354 (14·16% of TEI)	≤3·5% TEI	≥21% TEI	1
Added fat – saturated oils and *trans* fat (e.g., palm oil, coconut oil, dairy fat (butter), margarine, lard, tallow)	11·8 (0-11·8)	96 (3·8% of TEI)	≥10% TEI	0% TEI	1
Added sugar and sugar from fruit juice	31 (0-31)	120 (4·8% of TEI)	≥25% TEI	≤5% TEI	1

TEI: total energy intake;

© 2025 Yin X et al. *JAMA Network Open*.

eTable 2. Association Between PHDI and the Risk of T2D Among Women With a History of GD, Nurses' Health Study II

	**Quintiles of PHDI**					
	**Q1**	**Q2**	**Q3**	**Q4**	**Q5**	***P* - trend**
**T2D**						
T2D Cases (n=1053)	264	239	224	182	144	
Person-years	18570	18355	18117	17762	16915	
No of cases/10,000-person years	142	130	124	102	85	
Model 1 [a]	1.00 (reference)	0.84 (0.71-1.01)	**0.77 (0.65-0.93)**	**0.64 (0.53-0.78)**	**0.53 (0.43-0.65)**	**<0.001**
Model 2 [b]	1.00 (reference)	0.92 (0.77-1.10)	0.90 (0.75-1.08)	**0.78 (0.64-0.95)**	**0.70 (0.57-0.88)**	**<0.001**
Model 3 [c]	1.00 (reference)	0.95 (0.80-1.14)	0.95 (0.79-1.15)	0.86 (0.71-1.05)	0.88 (0.71-1.10)	0.16

Data were HRs (95% CIs) unless noted otherwise. Abbreviations: PHDI, Planetary Health Diet Index.

[a] Model 1 stratified by age (months) and calendar time.

[b] Model 2 additionally adjusted parity (1, ≥2), race/ethnicity (White/non-White), family history of diabetes/CVD (yes or no), oral contraceptive use (never, past, current user), menopausal status (premenopausal, postmenopausal, unsure), cigarette smoking (current, former, never), physical activity (continuous, MET-hours/week), total energy intake (continuous, kcal/d), alcohol intake (continuous, g/d), sodium intake (continuous, mg/d), ever had hypertension (yes or no), and ever had high cholesterol (yes or no).

[c] Model 3 additionally adjusted for BMI (continuous, kg/m^2).

© 2025 Yin X et al. *JAMA Network Open*.

eTable 3. Hazard Ratios (95% CIs) for Each Food Component of the PHDI for CVD and T2D Risk Among Women With a History of GD, Nurses' Health Study II

**Food group**	**Index (Score)**	**Index (Score)**	**Intake (Gram)**	**Intake (Gram)**	
	**Cumulative average score** T3 vs T1	**Cumulative average score** per 1 score (continuous; range 0-10)	**Cumulative average gram** T3 vs T1	**Cumulative average gram (continuous)** approximately scaled to the interval between 25th and 75th percentile	
**CVD**					
Whole grains	**0.50 (0.28-0.87)**	0.89 (0.78-1.02)	**0.52 (0.30-0.91)**	0.73 (0.51-1.05)	per 20 gram
Starchy vegetables*	**0.46 (0.24-0.89)**	**0.91 (0.82-0.99)**	**2.05 (1.08-3.89)**	1.28 (0.99-1.66)	per 60 gram
Vegetables	0.83 (0.47-1.45)	0.93 (0.84-1.03)	0.86 (0.49-1.48)	0.78 (0.58-1.06)	per 150 gram
Whole fruits	1.00 (0.58-1.71)	0.98 (0.90-1.06)	0.96 (0.56-1.65)	0.96 (0.72-1.27)	per 120 gram
Dairy*	1.14 (0.64-2.02)	1.05 (0.96-1.14)	0.88 (0.50-1.56)	0.83 (0.58-1.18)	per 400 gram
Red/processed meat*	0.97 (0.52-1.79)	0.95 (0.87-1.04)	1.30 (0.71-2.40)	1.19 (0.92-1.55)	per 50 gram
Poultry	1.60 (0.94-2.75)	1.03 (0.95-1.12)	0.72 (0.41-1.27)	0.88 (0.60-1.28)	per 50 gram
Eggs*	0.91 (0.55-1.50)	0.98 (0.80-1.20)	1.25 (0.72-2.17)	1.03 (0.89-1.18)	per 10 gram
Fish	0.66 (0.37-1.17)	0.95 (0.89-1.03)	0.51 (0.28-0.95)	**0.73 (0.56-0.96)**	per 20 gram
Nuts and seeds	0.69 (0.38-1.26)	0.96 (0.81-1.13)	0.64 (0.35-1.17)	1.10 (0.88-1.39)	per 10 gram
Legumes	1.18 (0.70-2.00)	1.03 (0.76-1.39)	1.17 (0.69-1.99)	1.01 (0.83-1.24)	per 15 gram
Soy†	0.61 (0.34-1.08)	1.05 (0.78-1.43)	0.61 (0.34-1.08)	1.00 (0.95-1.04)	per 5 gram
Added fat – Unsaturated oils (% of TEI)	1.16 (0.69-1.94)	1.01 (0.90-1.14)	1.20 (0.71-2.01)	1.06 (0.81-1.37)	per 5% of TEI
Added fat – Saturated oils, *trans* fat (% of TEI) *	0.65 (0.38-1.13)	0.93 (0.83-1.05)	1.46 (0.85-2.51)	1.35 (0.96-1.91)	per 5% of TEI
Added sugar and fruit juices (% of TEI) *	0.94 (0.56-1.59)	1.02 (0.93-1.11)	0.99 (0.58-1.67)	1.03 (0.73-1.45)	per 10% of TEI
- MI					
Whole grains	**0.40 (0.17-0.92)**	**0.77 (0.62-0.96)**	0.45 (0.20-1.01)	**0.51 (0.29-0.92)**	per 20 gram
Starchy vegetables*	0.47 (0.20-1.11)	**0.86 (0.76-0.98)**	1.72 (0.75-3.95)	1.37 (0.98-1.90)	per 60 gram
Vegetables	0.99 (0.45-2.17)	0.97 (0.84-1.12)	0.90 (0.41-1.99)	0.83 (0.55-1.25)	per 150 gram
Whole fruits	0.70 (0.32-1.52)	0.93 (0.83-1.04)	0.84 (0.40-1.78)	0.83 (0.55-1.25)	per 120 gram
Dairy*	1.49 (0.65-3.41)	**1.14 (1.00-1.29)**	0.67 (0.29-1.53)	0.59 (0.34-1.01)	per 400 gram
Red/processed meat*	0.60 (0.25-1.45)	0.87 (0.76-1.01)	2.10 (0.86-5.13)	1.22 (0.85-1.74)	per 50 gram

© 2025 Yin X et al. *JAMA Network Open*.

Poultry	1.34 (0.63-2.86)	1.02 (0.91-1.14)	0.79 (0.37-1.70)	0.97 (0.62-1.51)	per 50 gram
Eggs*	0.81 (0.41-1.61)	0.85 (0.69-1.05)	1.17 (0.55-2.48)	1.11 (0.94-1.31)	per 10 gram
Fish	0.53 (0.23-1.19)	0.94 (0.85-1.04)	**0.37 (0.15-0.91)**	0.73 (0.50-1.07)	per 20 gram
Nuts and seeds	0.53 (0.22-1.26)	1.00 (0.79-1.26)	0.53 (0.22-1.26)	1.05 (0.71-1.54)	per 10 gram
Legumes	1.46 (0.69-3.10)	1.10 (0.74-1.64)	1.45 (0.69-3.07)	1.06 (0.82-1.38)	per 15 gram
Soy†	0.53 (0.22-1.29)	0.70 (0.34-1.44)	0.53 (0.22-1.29)	0.91 (0.73-1.12)	per 5 gram
Added fat – Unsaturated oils (% of TEI)	1.06 (0.53-2.12)	1.00 (0.85-1.18)	1.06 (0.53-2.16)	0.97 (0.66-1.43)	per 5% of TEI
Added fat – Saturated oils, *trans* fat (% of TEI) *	0.84 (0.41-1.73)	1.01 (0.86-1.17)	0.97 (0.46-2.03)	1.09 (0.65-1.82)	per 5% of TEI
Added sugar and fruit juices (% of TEI) *	0.90 (0.45-1.79)	0.98 (0.87-1.10)	1.03 (0.51-2.05)	1.24 (0.85-1.80)	per 10% of TEI
- Stroke					
Whole grains	0.55 (0.27-1.14)	0.95 (0.80-1.13)	0.55 (0.27-1.14)	0.86 (0.55-1.34)	per 20 gram
Starchy vegetables*	0.47 (0.20-1.15)	0.96 (0.83-1.10)	2.10 (0.86-5.11)	1.17 (0.78-1.75)	per 60 gram
Vegetables	0.82 (0.39-1.74)	0.92 (0.81-1.06)	0.88 (0.42-1.82)	0.85 (0.57-1.28)	per 150 gram
Whole fruits	1.13 (0.55-2.31)	1.01 (0.90-1.14)	0.96 (0.47-1.97)	1.07 (0.73-1.57)	per 120 gram
Dairy*	1.21 (0.57-2.58)	1.01 (0.90-1.13)	0.85 (0.40-1.80)	0.96 (0.60-1.54)	per 400 gram
Red/processed meat*	1.24 (0.54-2.84)	0.99 (0.88-1.12)	1.01 (0.45-2.28)	1.26 (0.89-1.78)	per 50 gram
Poultry	2.11 (1.00-4.47)	1.07 (0.95-1.20)	0.58 (0.27-1.29)	0.77 (0.44-1.35)	per 50 gram
Eggs*	0.90 (0.46-1.78)	1.11 (0.77-1.60)	1.34 (0.63-2.85)	0.97 (0.78-1.22)	per 10 gram
Fish	0.90 (0.42-1.94)	0.97 (0.88-1.07)	0.65 (0.28-1.51)	0.74 (0.52-1.05)	per 20 gram
Nuts and seeds	0.70 (0.31-1.55)	0.98 (0.79-1.22)	0.70 (0.31-1.55)	1.22 (0.97-1.54)	per 10 gram
Legumes	1.07 (0.53-2.15)	1.01 (0.66-1.54)	1.06 (0.53-2.13)	0.99 (0.74-1.34)	per 15 gram
Soy†	0.70 (0.34-1.45)	1.23 (0.89-1.72)	0.70 (0.34-1.45)	1.01 (0.97-1.05)	per 5 gram
Added fat – Unsaturated oils (% of TEI)	1.06 (0.52-2.16)	0.99 (0.85-1.15)	1.12 (0.55-2.28)	1.06 (0.76-1.49)	per 5% of TEI
Added fat – Saturated oils, *trans* fat (% of TEI) *	0.66 (0.32-1.36)	0.93 (0.80-1.08)	1.69 (0.83-3.46)	1.33 (0.86-2.08)	per 5% of TEI
Added sugar and fruit juices (% of TEI) *	0.82 (0.38-1.74)	1.00 (0.88-1.14)	1.16 (0.55-2.44)	0.93 (0.55-1.57)	per 10% of TEI
**T2D**					
Whole grains	0.95 (0.81-1.12)	0.98 (0.95-1.02)	0.95 (0.81-1.12)	0.95 (0.86-1.04)	per 20 gram

© 2025 Yin X et al. *JAMA Network Open*.

Starchy vegetables*	1.04 (0.88-1.22)	1.00 (0.97-1.03)	1.04 (0.88-1.24)	0.98 (0.90-1.08)	per 60 gram
Vegetables	1.01 (0.86-1.19)	1.00 (0.97-1.03)	0.99 (0.84-1.16)	1.02 (0.94-1.10)	per 150 gram
Whole fruits	1.00 (0.85-1.18)	1.00 (0.98-1.03)	1.04 (0.88-1.23)	1.00 (0.92-1.08)	per 120 gram
Dairy*	0.95 (0.80-1.13)	0.99 (0.97-1.02)	1.01 (0.85-1.21)	1.03 (0.93-1.14)	per 400 gram
Red/processed meat*	**0.78 (0.66-0.93)**	**0.97 (0.94-0.99)**	1.18 (0.99-1.41)	1.08 (1.00-1.16)	per 50 gram
Poultry	1.05 (0.90-1.22)	1.01 (0.98-1.03)	0.96 (0.82-1.13)	0.97 (0.88-1.08)	per 50 gram
Eggs*	0.89 (0.77-1.03)	0.95 (0.89-1.01)	1.17 (0.99-1.37)	1.04 (1.00-1.09)	per 10 gram
Fish	0.95 (0.81-1.11)	1.00 (0.98-1.02)	0.97 (0.83-1.14)	1.02 (0.96-1.08)	per 20 gram
Nuts and seeds	1.06 (0.90-1.25)	1.00 (0.95-1.05)	1.06 (0.90-1.25)	0.98 (0.90-1.07)	per 10 gram
Legumes	1.01 (0.87-1.18)	1.02 (0.94-1.11)	1.03 (0.88-1.20)	1.02 (0.96-1.08)	per 15 gram
Soy†	**0.85 (0.73-1.00)**	0.91 (0.82-1.01)	**0.85 (0.73-1.00)**	1.00 (0.99-1.01)	per 5 gram
Added fat – Unsaturated oils (% of TEI)	0.95 (0.81-1.12)	0.97 (0.94-1.01)	0.90 (0.76-1.06)	0.93 (0.85-1.01)	per 5% of TEI
Added fat – Saturated oils, *trans* fat (% of TEI) *	0.94 (0.81-1.10)	0.99 (0.95-1.02)	1.05 (0.90-1.23)	1.05 (0.94-1.17)	per 5% of TEI
Added sugar and fruit juices (% of TEI) *	1.16 (1.00-1.36)	1.01 (0.98-1.04)	0.87 (0.74-1.01)	0.97 (0.87-1.07)	per 10% of TEI

TEI: total energy intake; Bold texts showed statistically significant HRs (*P*-value < 0.05).

All analyses were conducted using Cox models stratified by age (months) and calendar time. Multivariable model adjusted for parity (1, ≥2), race/ethnicity (White/non-White), family history of diabetes/CVD (yes or no), oral contraceptive use (never, past, current user), menopausal status (premenopausal, postmenopausal, unsure), cigarette smoking (current, former, never), physical activity (continuous, MET-hours/week), total energy intake (continuous, kcal/d), alcohol intake (continuous, g/d), sodium intake (continuous, mg/d), ever had hypertension (yes or no), ever had high cholesterol (yes or no), BMI (continuous, kg/m^2).

* A higher score represents lower consumption because the scores for these unhealthy food groups were reverse-coded.

† Given the limited variation in soy food consumption, the data was divided into two groups, and hazard ratios were calculated for intake versus non-intake.

© 2025 Yin X et al. *JAMA Network Open*.

eTable 4. Spearman Correlation Coefficients Between the Cumulative Mean PHDI and Other Dietary Patterns, Nurses' Health Study II

	PHDI	AHEI	AMED	Dash	PDI	hPDI	uPDI
**Correlation with PHDI, r_s**	-	0.84	0.64	0.49	0.27	0.59	-0.45
**Food component in each dietary pattern**							
Whole grains	√	√	√	√	√	√	√
Starchy vegetables	√				√	√	√
Vegetables	√	√	√	√	√	√	√
Whole fruits	√	√	√	√	√	√	√
Dairy food	√			√	√	√	√
Red/processed meat	√	√	√	√	√	√	√
Poultry	√				√	√	√
Eggs	√				√	√	√
Fish	√		√		√	√	√
Nuts & Seeds	√	√	√	√	√	√	√
Total Legumes	√	√	√	√	√	√	√
Non-soy Legumes	√						
Soybean	√						
Unsaturated oils	√	√	√		√	√	√
Saturated oils, *trans* fat	√	√	√		√	√	√
Sugar and fruit juices	√	√		√	√	√	√
Alcohol		√	√				
Sodium		√		√			

Abbreviations: PHDI, Planetary Health Diet Index; AHEI, Alternate Healthy Eating Index 2010; AMED, Alternate Mediterranean Diet Score; DASH, Dietary Approaches to Stop Hypertension Diet; PDI, Plant-based Diet Index; hPDI, Healthy Plant-based Diet Index; uPDI, Unhealthy Plant-based Diet Index.

© 2025 Yin X et al. *JAMA Network Open*.

eTable 5. Association Between PHDI and Risks of CVD and T2D Among Women With a History of GD, Excluding Cases in the First 4 Years of Follow-Up, Nurses' Health Study II

	Tertiles of PHDI			***P*** **-**
	**T1**	**T2**	**T3**	**trend**
**CVD**				
Model 1	1.00 (reference)	0.75 (0.46-1.25)	0.54 (0.30-0.95)	0.03
Model 2	1.00 (reference)	0.77 (0.47-1.28)	0.56 (0.32-0.99)	0.04
MI				
Model 1	1.00 (reference)	0.45 (0.22-0.92)	0.35 (0.15-0.81)	0.01
Model 2	1.00 (reference)	0.46 (0.23-0.94)	0.36 (0.15-0.84)	0.01
STOKE				
Model 1	1.00 (reference)	1.17 (0.57-2.42)	1.01 (0.48-2.15)	0.99
Model 2	1.00 (reference)	1.21 (0.59-2.50)	1.08 (0.51-2.32)	0.86
**T2D**				
Model 1	1.00 (reference)	0.97 (0.84-1.12)	0.79 (0.67-0.94)	0.01
Model 2	1.00 (reference)	1.03 (0.89-1.20)	0.92 (0.78-1.10)	0.41

[a] Full adjusted Model 1 stratified by age (months) and calendar time, and adjusted for parity (1, ≥2), race/ethnicity (White/non-White), family history of diabetes/CVD (yes or no), oral contraceptive use (never, past, current user), menopausal status (premenopausal, postmenopausal, unsure), cigarette smoking (current, former, never), physical activity (continuous, MET-hours/week), total energy intake (continuous, kcal/d), alcohol intake (continuous, g/d), sodium intake (continuous, mg/d), ever had hypertension (yes or no), ever had high cholesterol (yes or no).

[b] Model 2 additionally adjusted for BMI (continuous, kg/m^2).

© 2025 Yin X et al. *JAMA Network Open*.

eTable 6. Association Between PHDI and Risks of CVD and T2D Among Women With a History of GD and Available Sleep Duration (n = 3660), Nurses' Health Study II

	**Tertiles of PHDI**			***P*-trend**
	**T1**	**T2**	**T3**	
**CVD**				
Full adjusted Model [a]	1.00 (reference)	0.88 (0.51-1.49)	0.61 (0.33-1.12)	0.11
+ Sleep duration [b]	1.00 (reference)	0.87 (0.51-1.48)	0.61 (0.33-1.13)	0.12
MI				
Full adjusted Model [a]	1.00 (reference)	0.65 (0.31-1.36)	0.40 (0.16-1.04)	0.05
+ Sleep duration [b]	1.00 (reference)	0.65 (0.31-1.36)	0.40 (0.16-1.04)	0.05
STOKE				
Full adjusted Model [a]	1.00 (reference)	1.00 (0.48-2.11)	0.95 (0.44-2.06)	0.89
+ Sleep duration [b]	1.00 (reference)	1.00 (0.47-2.10)	0.95 (0.44-2.07)	0.89
**T2D**				
Full adjusted Model [a]	1.00 (reference)	0.97 (0.83-1.15)	0.88 (0.73-1.05)	0.17
+ Sleep duration [b]	1.00 (reference)	0.98 (0.83-1.15)	0.88 (0.73-1.05)	0.17

Data were HRs (95% CIs) unless noted otherwise. Abbreviations: PHDI, Planetary Health Diet Index.

[a] Full adjusted Model 1 stratified by age (months) and calendar time, and adjusted for parity (1, ≥2), race/ethnicity (White/non-White), family history of diabetes/CVD (yes or no), oral contraceptive use (never, past, current user), menopausal status (premenopausal, postmenopausal, unsure), cigarette smoking (current, former, never), physical activity (continuous, MET-hours/week), total energy intake (continuous, kcal/d), alcohol intake (continuous, g/d), sodium intake (continuous, mg/d), ever had hypertension (yes or no), ever had high cholesterol (yes or no), BMI (continuous, kg/m^2).

[b] Model 2 additionally adjusted for sleep duration (<7 hours, 7-8 hours, ≥9 hours).

© 2025 Yin X et al. *JAMA Network Open*.

eTable 7. Joint Association of PHDI and Physical Activity With Risks of CVD and T2D Among Women With a History of GD, Nurses' Health Study II

	Tertiles of PHDI		
	**T1**	**T2**	**T3**
**CVD**			
Physical activity (MET-hr/wk)			
<11.0 MET-hr/wk (median)	1.00 (reference)	0.62 (0.34-1.16)	0.54 (0.27-1.07)
≥11.0 MET-hr/wk (median)	0.59 (0.27-1.31)	0.77 (0.40-1.49)	0.43 (0.20-0.92)
MI			
Physical activity (MET-hr/wk)			
<11.0 MET-hr/wk (median)	1.00 (reference)	0.43 (0.18-1.01)	0.38 (0.13-1.06)
≥11.0 MET-hr/wk (median)	0.66 (0.25-1.74)	0.47 (0.17-1.29)	0.25 (0.07-0.90)
STOKE			
Physical activity (MET-hr/wk)			
<11.0 MET-hr/wk (median)	1.00 (reference)	0.85 (0.36-2.00)	0.89 (0.37-2.13)
≥11.0 MET-hr/wk (median)	0.51 (0.07-1.40)	0.91 (0.36-2.28)	0.69 (0.27-1.79)
**T2D**			
Physical activity (MET-hr/wk)			
<11.0 MET-hr/wk (median)	1.00 (reference)	0.96 (0.80-1.15)	1.06 (0.87-1.31)
≥11.0 MET-hr/wk (median)	0.89 (0.72-1.10)	0.97 (0.79-1.18)	0.72 (0.58-0.91)

Data were HRs (95% CIs) unless noted otherwise. Abbreviations: PHDI, Planetary Health Diet Index. Fully adjusted model stratified by age (months) and calendar time, and adjusted for parity (1, ≥2), race/ethnicity (White/non-White), family history of diabetes/CVD (yes or no), oral contraceptive use (never, past, current user), menopausal status (premenopausal, postmenopausal, unsure), cigarette smoking (current, former, never), total energy intake (continuous, kcal/d), alcohol intake (continuous, g/d), sodium intake (continuous, mg/d), ever had hypertension (yes or no), ever had high cholesterol (yes or no), BMI (continuous, kg/m^2).

© 2025 Yin X et al. *JAMA Network Open*.

eTable 8. Association Between 4-Year Change in PHDI and Least Squares Means of Weight Change (kg) Among Women With a History of GD, Nurses' Health Study II

**PHDI Changes by Tertile Transition Patterns**	**Weight Change, kg (95% CI)**	**Standardized Mean Differences (SMD)**
High to low	2.3 (1.6 - 3.0)	Reference
Median to low	2.1 (1.7 - 2.5)	0.025
Stay low	1.9 (1.6 - 2.2)	0.042
High to median	1.9 (1.5 - 2.3)	0.049
Stay medium	1.4 (1.1 - 1.6)	0.125
Low to median	1.2 (0.8 - 1.6)	0.144
Stay high	0.9 (0.6 - 1.3)	0.123
Median to high	0.4 (0.1 - 0.8)	0.269
Low to high	-0.3 (-1.0 - 0.4)	0.321
**PHDI Changes (Mean [SD]) by Quintiles**	**Weight Change, kg (95% CI)**	**Standardized Mean Differences (SMD)**
Quintile 1, -10.2 (6.1)	2.3 (2.0 - 2.6)	Reference
Quintile 2, -0.8 (1.2)	1.8 (1.5 - 2.0)	0.057
Quintile 3, 0.7 (1.2)	1.4 (1.1 - 1.7)	0.096
Quintile 4, 6.0 (3.0)	1.1 (0.9 - 1.4)	0.136
Quintile 5, 17.2 (6.9)	0.3 (0.0 - 0.5)	0.224

Abbreviations: GD, gestational diabetes; PHDI, planetary health diet index. Least squares (LS) means of 4-year weight change were modelled in multivariable marginal models with generalized estimating equations adjusting for follow-up period, age (continuous), parity (1, ≥2), race/ethnicity (White/non-White), family history of diabetes (yes or no), oral contraceptive use (never, past, current user), menopausal status (premenopausal, postmenopausal, unsure), cigarette smoking (current, former, never), physical activity (continuous, MET-hours/week), total energy intake (continuous, kcal/d), alcohol intake (continuous, g/d), sodium intake (continuous, mg/d), ever had hypertension (yes or no), ever had high cholesterol (yes or no), baseline PHDI values (continuous), and baseline BMI (continuous, kg/m^2).

© 2025 Yin X et al. *JAMA Network Open*.

eFigure 1. Association Between 4-Year Change in PHDI and Least Squares Means of Weight Change (kg) Among Participants ⩽65 Years, Nurses' Health Study II

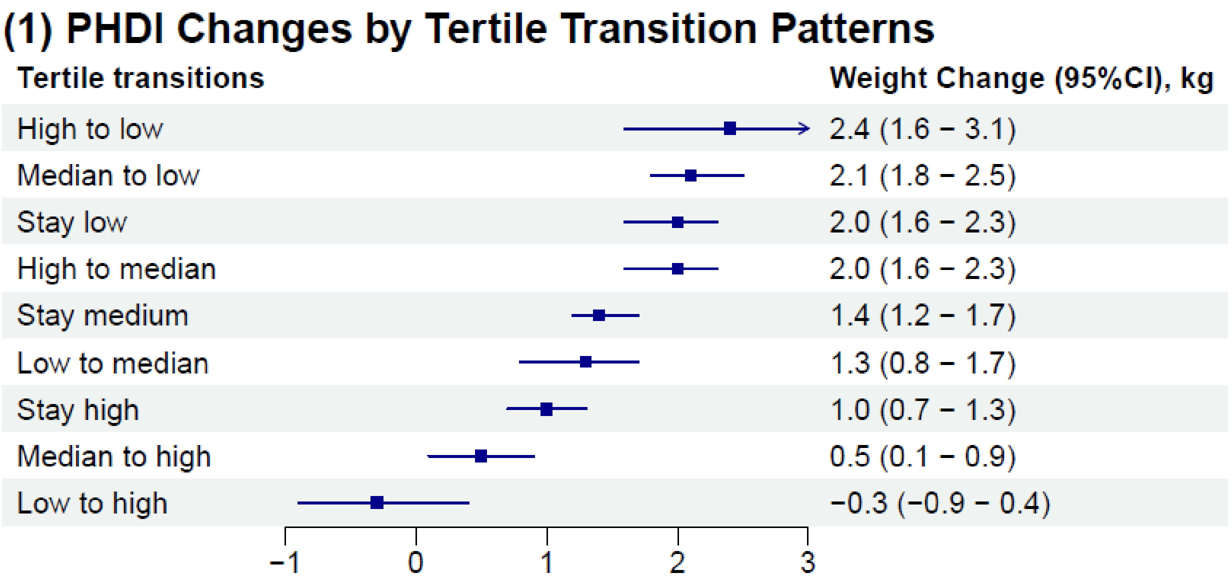

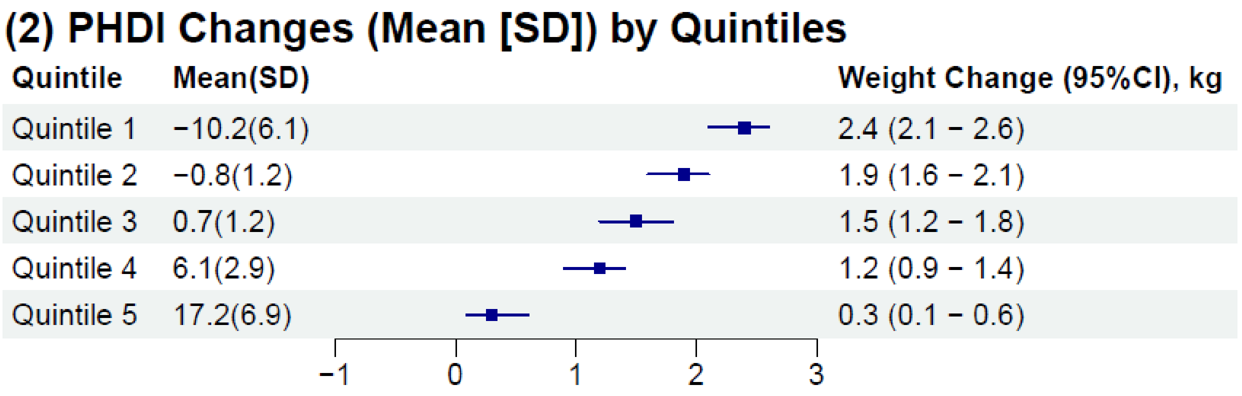

Abbreviations: GD, gestational diabetes; PHDI, planetary health diet index. Least squares (LS) means of 4-year weight change were modelled in multivariable marginal models with generalized estimating equations adjusting for follow-up period, age (continuous), baseline PHDI values (continuous), parity (1, ≥2), race/ethnicity (White/non-White), family history of diabetes (yes or no), oral contraceptive use (never, past, current user), menopausal status (premenopausal, postmenopausal, unsure), cigarette smoking (current, former, never), physical activity (continuous, MET-hours/week), total energy intake (continuous, kcal/d), alcohol intake (continuous, g/d), sodium intake (continuous, mg/d), ever had hypertension (yes or no), ever had high cholesterol (yes or no), and baseline BMI (continuous, kg/m^2).

© 2025 Yin X et al. *JAMA Network Open*.

eFigure 2. Cumulative Incidence of T2D and CVD by Tertiles of PHDI Among Women With a History of GD, Nurses' Health Study II

a. T2D

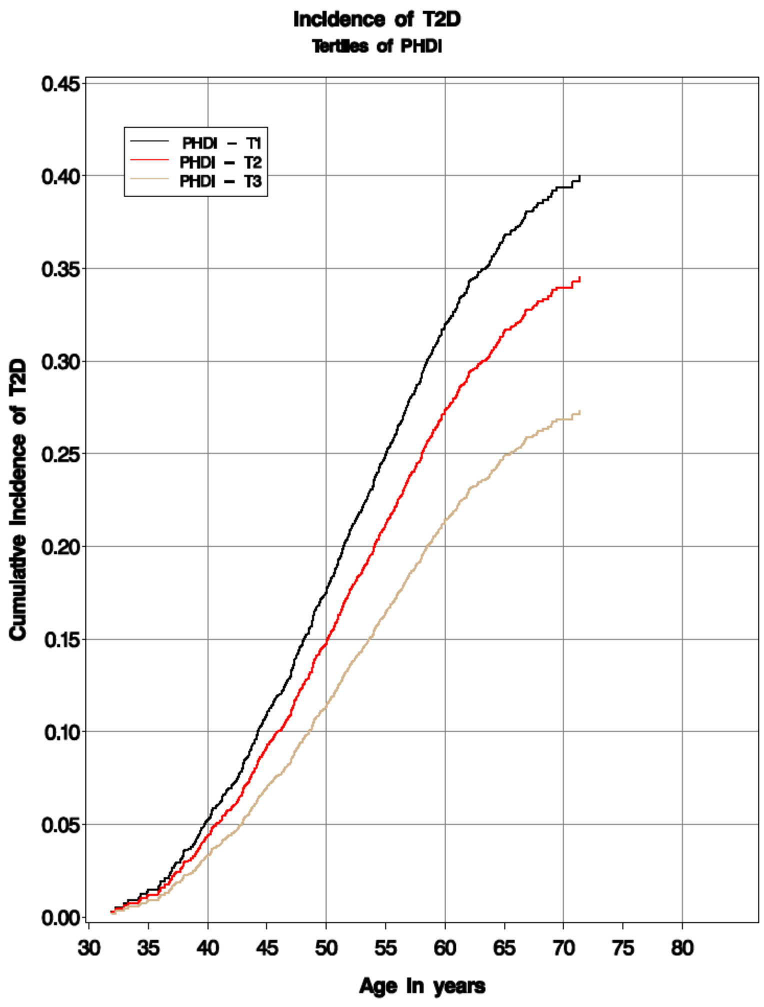

b. CVD

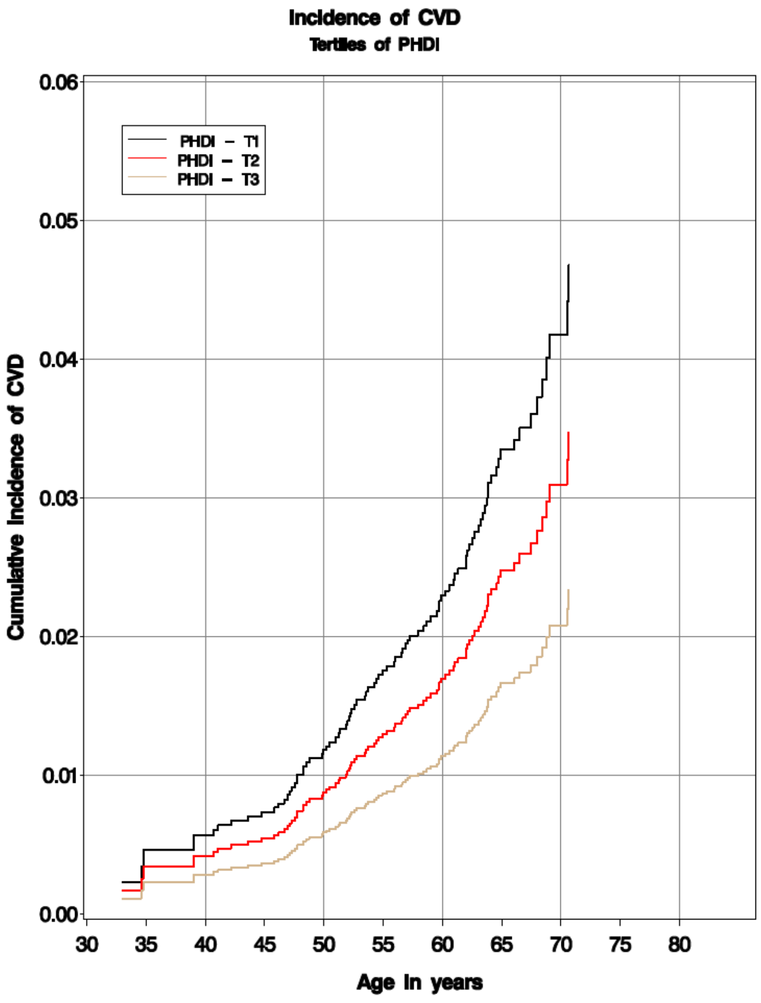

© 2025 Yin X et al. *JAMA Network Open*.

eReferences

1. Bao Y, Bertoia ML, Lenart EB, et al. Origin, Methods, and Evolution of the Three Nurses' Health Studies. *Am J Public Health*. Sep 2016;106(9):1573-81. doi:10.2105/ajph.2016.303338

2. Solomon CG, Willett WC, Carey VJ, et al. A prospective study of pregravid determinants of gestational diabetes mellitus. *JAMA*. Oct 1 1997;278(13):1078-83.

3. Willett WC, Reynolds RD, Cottrell-Hoehner S, Sampson L, Browne ML. Validation of a semi-quantitative food frequency questionnaire: comparison with a 1-year diet record. *J Am Diet Assoc*. Jan 1987;87(1):43-7.

4. Expert Committee on the Diagnosis and Classification of Diabetes Mellitus. Report of the Expert Committee on the Diagnosis and Classification of Diabetes Mellitus. *Diabetes Care*. Jul 1997;20(7):1183-97.

5. Group NDD. Classification and Diagnosis of Diabetes Mellitus and Other Categories of Glucose Intolerance. *Diabetes*. 1979;28(12):1039-1057. doi:10.2337/diab.28.12.1039

6. Mendis S, Thygesen K, Kuulasmaa K, et al. World Health Organization definition of myocardial infarction: 2008-09 revision. *International journal of epidemiology*. Feb 2011;40(1):139-46. doi:10.1093/ije/dyq165

7. Walker AE, Robins M, Weinfeld FD. The National Survey of Stroke. Clinical findings. *Stroke*. Mar-Apr 1981;12(2 Pt 2 Suppl 1):I13-44.

8. Stampfer MJ, Willett WC, Speizer FE, et al. Test of the National Death Index. *American journal of epidemiology*. May 1984;119(5):837-9. doi:10.1093/oxfordjournals.aje.a113804

9. Gaddey HL, Holder K. Unintentional weight loss in older adults. *Am Fam Physician*. May 1 2014;89(9):718-22.

10. Sun X, Du T. Trends in weight change patterns across life course among US adults, 1988–2018: population-based study. *BMC Public Health*. 2023/11/06 2023;23(1):2168. doi:10.1186/s12889-023-17137-x

11. Chen C, Ye Y, Zhang Y, Pan XF, Pan A. Weight change across adulthood in relation to all cause and cause specific mortality: prospective cohort study. *Bmj*. Oct 16 2019;367:l5584. doi:10.1136/bmj.l5584

© 2025 Yin X et al. *JAMA Network Open*.

12. Wolf AM, Hunter DJ, Colditz GA, et al. Reproducibility and validity of a self-administered physical activity questionnaire. *International journal of epidemiology*. Oct 1994;23(5):991-9.

13. Lin DY, Fleming TR, De Gruttola V. Estimating the proportion of treatment effect explained by a surrogate marker. *Stat Med*. Jul 15 1997;16(13):1515-27. doi:10.1002/(sici)1097-0258(19970715)16:13<1515::aid-sim572>3.0.co;2-1

14. Jun HJ, Austin SB, Wylie SA, et al. The mediating effect of childhood abuse in sexual orientation disparities in tobacco and alcohol use during adolescence: results from the Nurses' Health Study II. *Cancer Causes Control*. Nov 2010;21(11):1817-28. doi:10.1007/s10552-010-9609-3

15. Tobias DK, Hu FB, Chavarro J, Rosner B, Mozaffarian D, Zhang C. Healthful dietary patterns and type 2 diabetes mellitus risk among women with a history of gestational diabetes mellitus. *Arch Intern Med*. Nov 12 2012;172(20):1566-72. doi:10.1001/archinternmed.2012.3747

16. Harrell F. *Regression Modeling Strategies: With Applications to Linear Models, Logistic and Ordinal Regression, and Survival Analysis*. 2015.

17. Yin X, Bao W, Ley SH, et al. Sleep Characteristics and Long-Term Risk of Type 2 Diabetes Among Women With Gestational Diabetes. *JAMA Network Open*. 2025;8(3):e250142-e250142. doi:10.1001/jamanetworkopen.2025.0142

18. Chiuve SE, Fung TT, Rimm EB, et al. Alternative dietary indices both strongly predict risk of chronic disease. Research Support, N.I.H., Extramural
Research Support, Non-U.S. Gov't. *The Journal of nutrition*. Jun 2012;142(6):1009-18. doi:10.3945/jn.111.157222

19. Fung TT, Chiuve SE, McCullough ML, Rexrode KM, Logroscino G, Hu FB. Adherence to a DASH-style diet and risk of coronary heart disease and stroke in women. *Arch Intern Med*. Apr 14 2008;168(7):713-20. doi:10.1001/archinte.168.7.713

20. Trichopoulou A, Costacou T, Bamia C, Trichopoulos D. Adherence to a Mediterranean diet and survival in a Greek population. *N Engl J Med*. Jun 26 2003;348(26):2599-608. doi:10.1056/NEJMoa025039

21. Musicus AA, Wang DD, Janiszewski M, et al. Health and environmental impacts of plant-rich dietary patterns: a US prospective cohort study. *The Lancet Planetary Health*. 2022;6(11):e892-e900. doi:10.1016/S2542-5196(22)00243-1

© 2025 Yin X et al. *JAMA Network Open*.
