# Supplementary material for: Planetary Health Diet and Risk of Cardiometabolic Diseases Among Women With Gestational Diabetes
Source: JAMA Netw Open. 2025 Nov 7;8(11):e2540170. doi: 10.1001/jamanetworkopen.2025.40170 (PMC12595533; doi:10.1001/jamanetworkopen.2025.40170)
Supplement: Supplement 2. — Data Sharing Statement [file jamanetwopen-e2540170-s002.pdf]

Data Sharing Statement

Yin. Planetary Health Diet and Risk of Cardiometabolic Diseases Among Women With Gestational Diabetes. *JAMA Netw Open*. Published November 07, 2025. doi:10.1001/jamanetworkopen.2025.40170

Data
Data available: No

Additional Information
Explanation for why data not available: Statistical analysis codes used for the present analysis can be made available on a case-by-case basis with approval from the senior author of this manuscript. Data described in the manuscript will not be made publicly available. Further information including the procedures for obtaining and accessing data from the Nurses' Health Studies II is described online (https://www.nurseshealthstudy.org/researchers; email nhsaccess@channing.harvard.edu).
